# Illumination and gaze effects on face evaluation: The Bi-AGI database

**DOI:** 10.3389/fpsyg.2022.948142

**Published:** 2022-10-13

**Authors:** Giulia Mattavelli, Irene Gorrino, Elisabetta Cesana, Jacopo De Angelis, Paola Ricciardelli

**Affiliations:** ^1^IUSS Cognitive Neuroscience (ICoN) Center, Scuola Universitaria Superiore IUSS, Pavia, Italy; ^2^Istituti Clinici Scientifici Maugeri IRCCS, Cognitive Neuroscience Laboratory of Pavia Institute, Pavia, Italy; ^3^Department of Psychology, Università di Milano-Bicocca, Milan, Italy; ^4^Milan Centre for Neuroscience, Milan, Italy

**Keywords:** face evaluation, gaze direction, age, illumination, face database

## Abstract

Face evaluation and first impression generation can be affected by multiple face elements such as invariant facial features, gaze direction and environmental context; however, the composite modulation of eye gaze and illumination on faces of different gender and ages has not been previously investigated. We aimed at testing how these different facial and contextual features affect ratings of social attributes. Thus, we created and validated the Bi-AGI Database, a freely available new set of male and female face stimuli varying in age across lifespan from 18 to 87 years, gaze direction and illumination conditions. Judgments on attractiveness, femininity-masculinity, dominance and trustworthiness were collected for each stimulus. Results evidence the interaction of the different variables in modulating social trait attribution, in particular illumination differently affects ratings across age, gaze and gender, with less impact on older adults and greater effect on young faces.

## Introduction

Face perception is a widely explored human ability in experimental psychology. Faces convey crucial information for social interaction and humans are highly skilled in face processing. Indeed, few milliseconds of exposure are sufficient to detect and evaluate facial features (Bar et al., 2006; Willis and Todorov, 2006) and neuroimaging studies suggest that brain areas in the occipital and temporal cortices are specifically deputed to face processing (Haxby et al., 2000; Ishai, 2008). Face images are stimuli commonly used in many studies investigating specific aspects of face processing, or addressing questions related to social interaction and attention. Yet, the appearance of the same face can change from one image to another in terms of pictures properties and facial characteristics: for example, lighting, head rotation and expression can vary, while the features of the camera (e.g., focal length, shutter, lens settings) influence the overall image quality (Burton, 2013). The interplay between these sources of variation contributes to the development of subsequent impressions, which may actually diverge between images of the same face (Todorov and Porter, 2014).

To address researchers’ needs for face stimuli varied in different features, many databases are available in the literature (a collection of references and links could be found at www.face-rec.org). Facial expression is one of the most extensively varied condition in databases presenting models posing different emotions (e.g., FEEST set, Young et al., 2002; KDEF, Lundqvist et al., 1998; Bosphorus database, Savran et al., 2008; Radboud Faces Database, Langner et al., 2010). Most databases have young/middle-age models portrayed with direct gaze, but there are also available stimuli sets with models of different ages across lifespan (Minear and Park, 2004; O’Reilly et al., 2016) and the FACES-database (Ebner et al., 2010) included young, middle-age and older models posing six different emotions. Age is indeed a crucial dimension in face processing, it is linked with attractiveness (Thornhill and Gangestad, 1999; Perrett et al., 2002) and face preference (Ebner, 2008; Ito et al., 2022), it correlates with perceived height, masculinity and dominance (Batres et al., 2015), and a same-age effect is reported for face recognition (Bäckman, 1991); namely, better performances in recognizing individuals similar to own age. Moreover, a recent factor analysis carried out to model the structure of perception of personality traits from highly variable face stimuli (Sutherland et al., 2013) resulted in a three-dimensional model with approachability, dominance and youthful-attractiveness as factors predicting face evaluation. These studies showed that age is a relevant dimension in face processing, thus its impact should be considered in studying human face perception.

Another critical aspect perceived from faces during human interaction is gaze direction. In light of its essential social function, the ability to process gaze cues has been extensively studied in neuroimaging, developmental, and social cognition research (Allison et al., 2000; Emery, 2000; Frischen et al., 2007). Several studies have shown that gaze direction affects facial expression processing (Adams and Kleck, 2003, 2005; Sander et al., 2007; Milders et al., 2011) and social judgments (Mason et al., 2005; Bayliss and Tipper, 2006; Mattavelli et al., 2021). In particular, faces with gaze directed to the perceivers are rated as more dominant (Main et al., 2009), more attractive and trustworthy (Ewing et al., 2010; Kaisler and Leder, 2016; Mattavelli et al., 2021) than faces with averted eyes. Eye gaze is also an effective cue to orient visuospatial attention towards the same direction where other people are attending, a phenomenon known as joint attention (Driver et al., 1999). In this regard, different experiments have explored joint attention in healthy and clinical populations by means of the gaze-cueing paradigm, namely tasks in which participants are asked to detect or discriminate a target presented laterally to a face gazing congruently or incongruently to the region in which the target will appear (Frischen et al., 2007; Mattavelli et al., 2021). Faces with averted gaze direction have been also used in studies exploring attentional resources and memory processes (Frischen and Tipper, 2004, 2006; Artuso et al., 2012; Ricciardelli et al., 2012), and investigating the impact of emotion and gaze cue on the attribution of social traits to faces (Manssuer et al., 2015a,b; Mattavelli et al., 2021). Despite this wide use of faces with manipulated eye gaze in different experimental paradigms, most authors create their own gaze-cue stimuli with image editing software, which is a time-consuming procedure for researchers and the final effect could be not completely naturalistic. Few databases portrayed faces with right- and left-directed eye gaze. Specifically, the Radboud Faces Database (Langner et al., 2010) includes images of adults and children with eight emotional expressions and three gaze directions; Weidenbacher et al. (2007) presented a database of 20 individuals with various combinations of head pose and eye gaze; the ADFES by Van Der Schalk et al. (2011) includes young adults with different expression and head orientation. However, to the best of our knowledge there are no databases of face stimuli with different gaze directions covering different ages across lifespan, and such a stimulus set would be a valuable resource for future work examining in greater detail how perceived age modulates gaze following behaviour (see Ciardo et al., 2014).

Moving from features of the face to contextual variables, it has been shown that within-person variability among different pictures has a critical impact on face processing, in particular when ambient images are employed, namely pictures of naturally occurring faces with surrounding environment (Burton, 2013). Face judgments in terms of attractiveness or social attribution as trustworthiness, competence and intelligence can show within-person variability as much as between-person variability, and when different ambient pictures of the same face are presented for matching tasks, they are often wrongly sorted as belonging to different people (Jenkins et al., 2011; Todorov and Porter, 2014). Previous studies have investigated the effect of changing in illumination condition on face recognition (Hill and Bruce, 1996; Braje et al., 1998; Braje, 2003). Indeed, illumination can affect the amount and/or type of information gathered from faces, with impact on human visual processing, including colour perception (e.g., Shin et al., 2004; Kelber et al., 2017), visual acuity (e.g., Sheedy et al., 1984; Ferwerda, 1998; Hiraoka et al., 2015) and contrast sensitivity (e.g., Amesbury and Schallhorn, 2003; Alghwiri and Whitney, 2012; Wood, 2020). Consistently, studies on facial memory tests suggested an overall negative impact on face identification when illuminated by dimmer light (DiNardo and Rainey, 1989; Nyman et al., 2019; Lim et al., 2022). Moreover, lighting is accurately controlled in professional photography, different light effects are used to emphasize face properties in portraits (Arena, 2012), and specific methods are applied to manipulate and digitally correct the desired effects (Shu et al., 2017). Two examples of widely used artistic effects in portraits are the Rembrandt and split styles, which create particular shadows under the eye or on half face, respectively (Jin et al., 2010). Interestingly, the Rembrandt effect derives from the style of the famous Dutch painter who made portraits with the same features, and it has been shown that such particular position and lighting could impact aesthetic/social judgments and emotional expressions with differences related to hemispheric laterality and gender of faces (Schirillo, 2000, 2014; Powell and Schirillo, 2011). These studies opened interesting questions on the relationship between illumination effects, that can be created with professional photography, and face evaluation. Some face databases are available with pictures taken with different illumination conditions (e.g., Martinez and Benavente, 1998; Georghiades et al., 2001; Sim et al., 2003; Gao et al., 2004), however they do not report the effect of this variable on face evaluation. Crucially, the impact of illumination conditions on different age and gender faces requires further investigation.

The different research areas on face perception reviewed above, evidence that face judgments result from the composite processing of different facial features and contextual factors. In order to investigate the possible combined influence of age, gaze direction and photographic effects on face judgment and person perception (Fiske et al., 2007). We developed a new set of stimuli evaluated for different social dimensions. We present here the University of Milano-Bicocca Age, Gaze and Illumination (Bi-AGI) database, a new set of face images with male and female models of wide age range, portrayed with three different photographic lighting conditions and three gaze directions. Stimuli were rated on the social dimensions of trustworthiness, dominance, attractiveness and femininity-masculinity. The study aimed at providing a high-quality set of face stimuli for future studies in face perception, social cognition and cognitive and social neuroscience. The ratings collected for stimuli validation were also analysed to empirically explore how the different facial features such as gaze direction, gender and age combined with different illumination conditions affect the perception of social attributes.

## Materials and methods

### Image set and apparatus

The database comprises 270 portrait images of 30 Caucasian models selected from three age ranges with five males and five females in each subgroup: young adults (mean age = 22.29, *sd* = 2.91, range 18–25 years), middle-age adults (mean age = 38.84, sd = 3.55, range 35–45 years), older adults (mean age = 72.16, *sd* = 11.7, range 55–87 years). We considered the criterion >55 years old for the older adults age class, to provide the database with stimuli covering a wide age range across lifespan. All models were asked to maintain a neutral expression while they were portrayed nine times: three gaze directions (direct, right-oriented, left-oriented) each in three illumination conditions (flat, Rembrandt, split). Models were asked not to wear glasses or hat, but no other constraints were specified for hairdos, make up or earrings to maintain natural occurring variance in face images. The study was approved by the Ethic Committee of the University Milano-Bicocca and all models signed informed consent and consented to the use of their images for experimental research.

**Figure 1:**
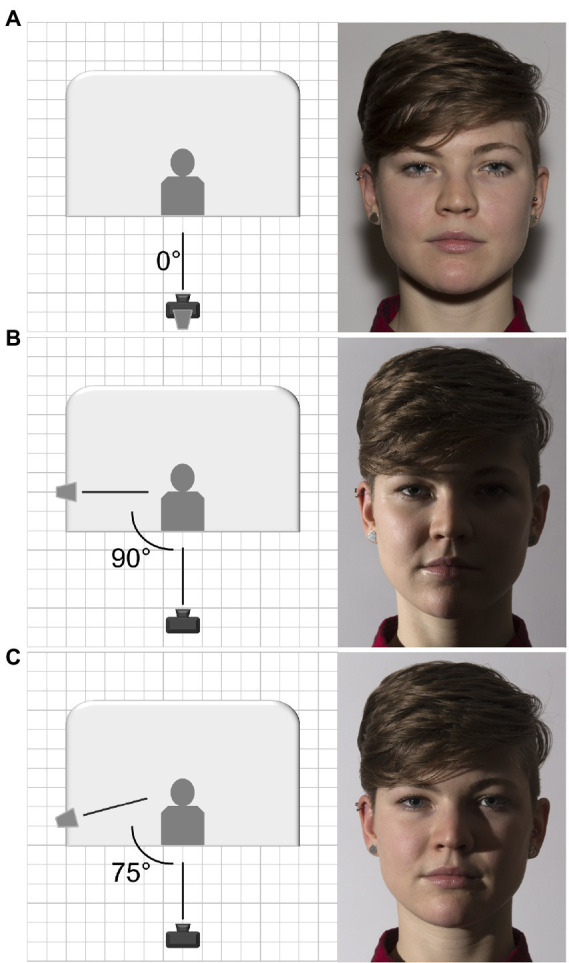
Photograph setting and example of one image for the flat **(A)**, split **(B)**, and Rembrandt **(C)** lighting conditions.

High-quality digital photographs were taken with a professional camera digital Reflex Canon Eos 6D with a full-frame sensor, a 1:1 lens crop factor and a Sigma 24–60 mm f/2.8 lens assembled on the camera body. The camera was fixed on a tripod while models stood against a white opaque background. The eye gaze was manipulated asking models to look towards the camera or above their left or right shoulder without turning the head. Lighting conditions were obtained by means of an external Nissin D622 flash assembled on the body camera or separated as a lateral flashing light triggered by a wireless Yongnuo RF-603C II. In the flat condition, the flash was on the body camera together with a softbox to create a uniform and natural light diffusion (see Figure 1A). In the split condition, the external flash was located at 90° on the right side of the models producing a darkened effect on the left side of model face (see Figure 1B). In the Rembrandt condition, the external flash was located at 75° on the right side of the models producing a partial darkness on the model face with the characteristic illuminated triangle under the eye (see Figure 1C). All the pictures included in the database are in color mode RGB with a 72 × 72 ppi resolution.

### Stimuli validation and statistical analyses

One hundred thirty-five volunteers (69 females, 66 males, mean age 42.79, *sd* = 16.62, range 20–86 years, mean education = 13.33, *sd* = 3.37) participated in the validation study. Ratings for four socially relevant dimensions were collected: trustworthiness, dominance, attractiveness and femininity-masculinity. These ratings were presented in four separated blocks in counterbalanced order across participants; within each block the 270 images were presented in random order. Data were collected using E-prime software (Psychology Software Tools, Pittsburgh, PA, USA). The images, resized to 170 × 232 pixels, were presented at the center of the screen with below the 7-point Likert scale with labels of the two extremes of the dimension above the number 1 and number 7 (not trustworthy at all–very trustworthy; not dominant at all–very dominant; not attractive at all–very attractive; very feminine–very masculine). The participants were asked to rate each image pressing the corresponding button from 1 to 7.

Furthermore, since we asked the model to pose with a neutral expression, we were interested to verify whether faces were actually perceived as expressing neutral emotion. Thus, a different sample of 30 healthy participants (15 females, age range 18–30 years old) was involved in an emotion recognition task. Only face stimuli with direct gaze were presented in the three illumination conditions for a total of 90 trials. Stimuli were presented at the center of the screen with below the seven options of emotional expression (neutral, surprise, happiness, fear, disgust, anger, sadness) and the corresponding button to press (from 1 to 7). The order of the emotional labels was counterbalanced across participants, who were asked to press the button corresponding to the expression of the presented faces.

Statistical analyses were performed in R programming environment (R Development Core Team, 2008) throughout R studio (version 2022.2.3.492). Rating scores for attractiveness, femininity-masculinity, dominance and trustworthiness were submitted to a series of linear mixed-effects regressions using the LMER procedure (Baayen et al., 2008), introducing illumination (flat, Rembrandt, split), gaze (central, averted), gender (male, female), and age (young, middle-age, older adults) of face stimuli as fixed factors, while the random-effects structure included by-subject (i.e., participants who rated the stimuli) and by-model (i.e., identity of face images) intercepts to account for inter-subject and inter-stimuli variability. In line with previous experiments (Mattavelli et al., 2021), evaluations of faces looking to the right- or left-side were considered as a unique ‘averted’ level compared to ‘central’ gaze to assess the overall impact of faces looking at (*vs* away from) the observer. Likelihood ratio tests were used to evaluate whether the introduction of the *fixed factors* and random effects significantly increased the models’ goodness of fit (Gelman and Hill, 2006), then only factors significant as main effect, or in interaction with other factors, were included in the final model. The “phia” R package (version 0.2–1, De Rosario-Martinez, 2015) was used for *post-hoc* pairwise contrasts on significant main effects and interactions on the final best-fitting models, applying Bonferroni correction for multiple comparisons. For the sake of simplicity, we report in the results section for each rating the significant main effects and higher order interactions looking at contrasts between illumination conditions. Tables on model selection and *post-hoc* contrasts are reported in Supplementary material. Appendix A includes mean rating values for each model separately.

## Results

### Control experiment on emotional expression

The aim of the experiment was to check if faces included in the database were perceived with a neutral expression. To do this, we compared participant responses (i.e., RESPs) with an expected distribution of equiprobability. Specifically, the equal distribution of the responses would show that participants attributed an emotional value to the faces rather than a neutral one. Conversely, a skewed distribution, with a greater frequency of neutral responses, would show a greater tendency of the participants to attribute a neutral valence to faces. Thus, a Chi-Square test of goodness was applied to determine whether RESPs distribution was likely to come from a distribution where RESPs were equally distributed or not – given a total of 7 RESPs the equal distribution would result into a 14.28% of responses for each emotion.

All the assumptions of the Chi-Square test were met and the test was applied by setting the threshold for statistical significance at 0.05. The results of the test led to reject the null hypothesis of equal distribution and to accept the alternative one supporting a greater frequency for specific RESPs categories (*χ*^2^(6) = 1121.108, *p* < 0.0001). Responses distribution is shown in the Supplementary Table S1. This shows an asymmetry towards neutral responses (30.4%) and none of the stimuli was excluded from the database on the basis of emotional expression control experiment. All the percentage values corresponding to emotional categories are indeed below 14.28% except for happiness (26.6%). Similar results were obtained by applying a binomial test to each neutral-emotional RESPs pair (Supplementary Table S2) and setting the equal distribution percentage at 50%. As in the case of the chi-squared results, the percentage of neutral responses is significantly higher than “emotional” responses except for the case of happiness.

### Attractiveness

**Figure 2:**
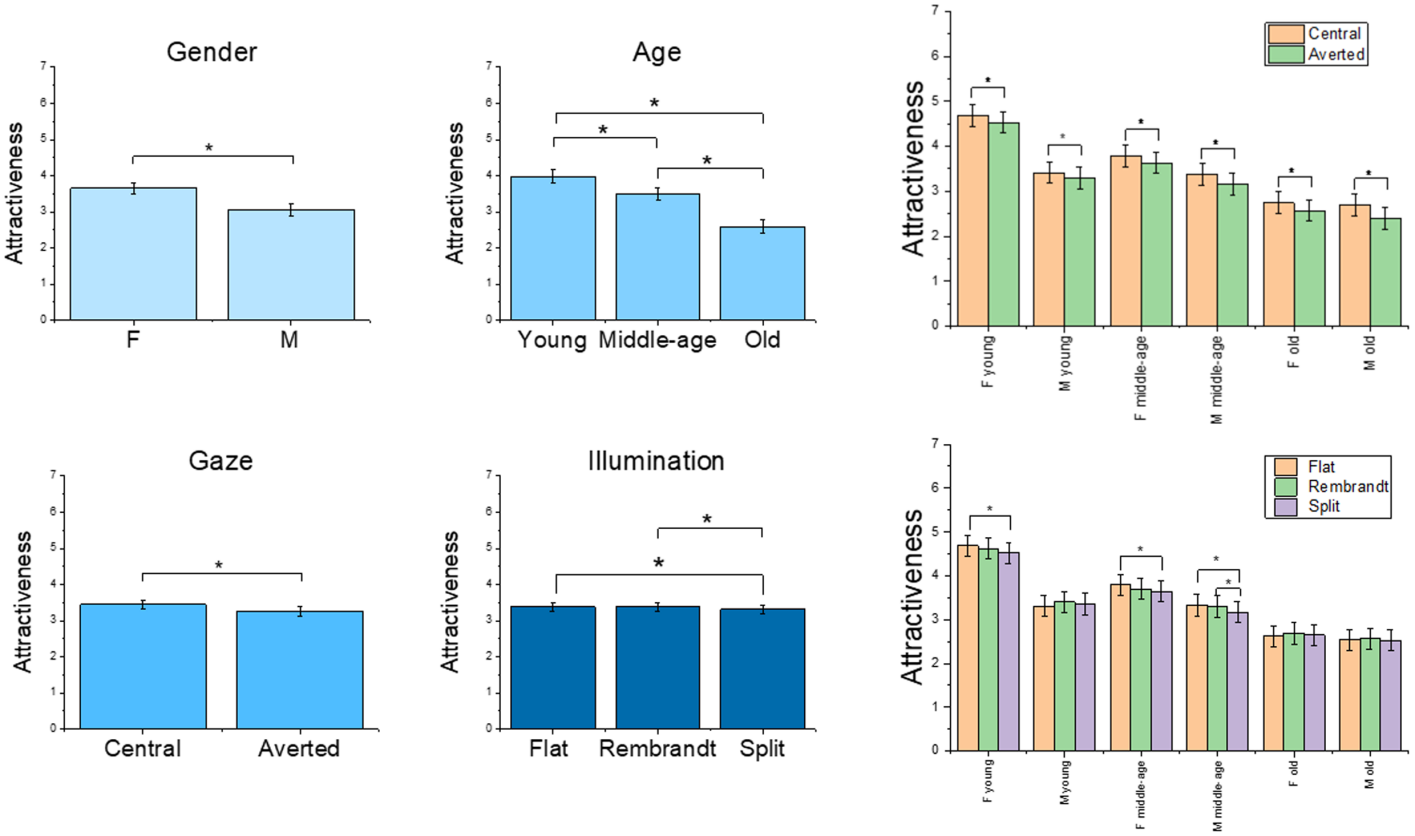
Plot of the estimated mean scores from the attractiveness rating, depicting significant main effects and higher order interactions. The asterisks highlight significant main effects and significant results from the *post hoc* analyses. C = central gaze, A = averted gaze, F = female models, M = male models. Error bars represent mean standard errors.

The final model on the attractiveness rating included the main effects of illumination [*χ*^2^(2) = 23.33, *p* < 0.001], gender [*χ*^2^(1) = 10.98, *p* < 0.001], age [*χ*^2^(2) = 39.68, *p* < 0.001] and gaze [*χ*^2^(1) = 169.41, *p* < 0.001] as well as the three-way interactions illumination x gender x age [*χ*^2^(4) = 12.7, *p* = 0.012] and gender x age x gaze [*χ*^2^(2) = 6.19, *p* = 0.045] (see Supplementary Table S3 for the model selection). Faces with central gaze were rated as more attractive than faces with averted gaze, and females received higher scores than males. *Post-hoc* tests on main effects showed that flat and Rembrandt illumination received higher score than split illumination (*ps* < 0.001), whereas young models received higher scores than middle age (*p* = 0.03) and old adults (*p* < 0.001), and middle age received higher scores than older adults (*p* < 0.001). *Post-hoc* comparison for the interaction illumination x gender x age showed that split illumination reduced attractiveness score compared to flat illumination in young (*p* < 0.001) and middle age (*p* = 0.002) female models, whereas middle age male models received lower scores for split compared to both flat (*p* = 0.001) and Rembrandts (*p* = 0.02) illumination. Pairwise contrasts on central vs. averted gaze for the gender x age x gaze interaction, revealed significant higher score for central gaze in male and female models of all age classes (all *ps* < 0.001) (see Figure 2).

### Femininity-masculinity

**Figure 3:**
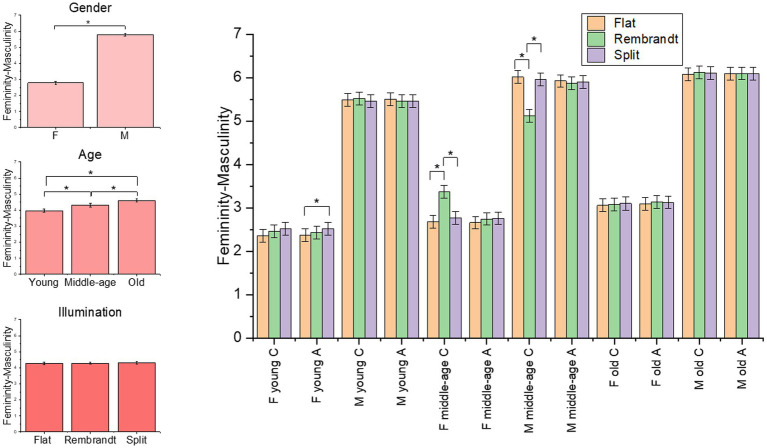
Plot of the estimated mean scores from the femininity-masculinity rating, depicting significant main effects and higher order interaction. The asterisks highlight the significant main effect and significant results form *post hoc* analyses. C = central gaze, A = averted gaze, F = female models, M = male models. Error bars represent mean standard errors.

The final model on the femininity-masculinity rating included the four-way interaction [*χ*^2^(4) = 154.14, *p* < 0.001]. The main effects of illumination [*χ*^2^(2) = 6.03, *p* = 0.049], gender [*χ*^2^(1) = 726.17, *p* < 0.001] and age [*χ*^2^(2) = 21.87, *p* < 0.001] were significant (see Supplementary Table S4 for the model selection). As expected, females were clearly rated as more feminine than male models, moreover *post-hoc* tests for illumination main effect revealed a trend for a higher masculine evaluation of models with split than flat illumination (*p* = 0.06) and *post-hoc* on age effect revealed that older adults were rated as more masculine than middle-age (*p* = 0.03) and young adults (*p* < 0.001), and middle-age were rated as more masculine than young adults (*p* = 0.02). Contrasts on the four-ways interaction showed that illumination impacted scores of male and female middle-age models with central gaze, since Rembrandt condition increased masculinity score of females (i.e., females appeared less feminine), and oppositely decreases masculinity score of males (i.e., males appeared less masculine), compared to flat and split conditions (all *ps* < 0.001). Moreover, in the case of averted gaze, female young models were rated as more masculine with split compared to flat illumination (*p* = 0.045) (see Figure 3).

### Dominance

**Figure 4:**
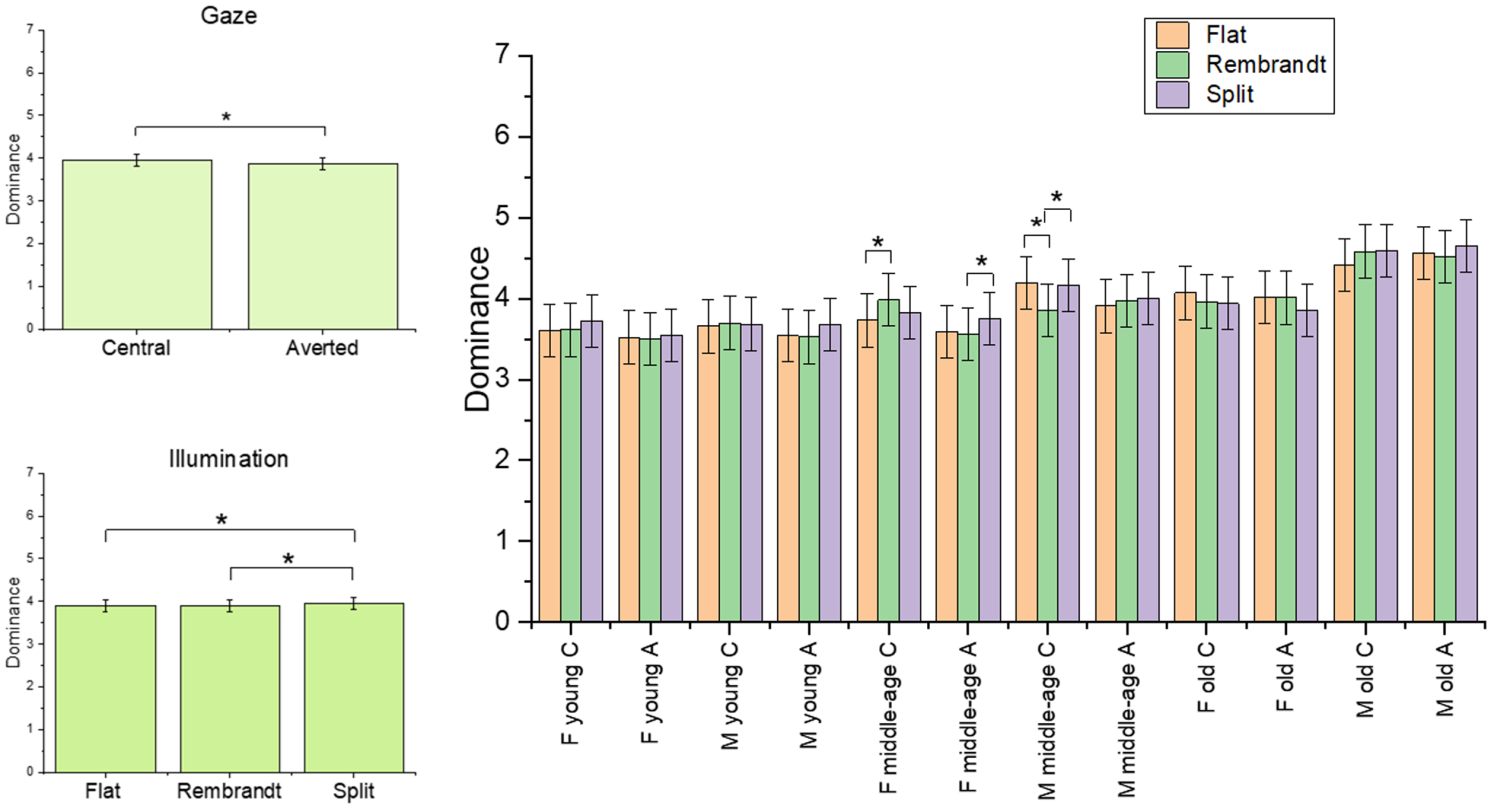
Plot of the estimated mean scores from the dominance rating, depicting significant main effects and higher order interaction. The asterisks highlight the significant main effect and significant results form *post hoc* analyses. C = central gaze, A = averted gaze, F = female models, M = male models. Error bars represent mean standard errors.

The final model on the dominance rating included the four-way interaction [*χ*^2^(4) = 40.63, *p* < 0.001]. The main effects of illumination [*χ*^2^(2) = 11.58, *p* = 0.003] and gaze [*χ*^2^(1) = 30.99, *p* < 0.001] were significant (see Supplementary Table S5 for the model selection). Faces with central gaze were rated more dominant, and scores increased for split compared to flat (*p* = 0.02) and Rembrandt (*p* = 0.02) illumination. *Post-hoc* analyses on the interaction revealed that in middle-age female models, Rembrandt illumination increased dominance scores compared to flat illumination in case of central gaze (*p* = 0.03), whereas split illumination increased dominance compared to Rembrandt illumination in case of averted gaze (*p* = 0.01). On the other hand, male middle-age models with central gaze were rated more dominant with flat and split illumination compared to Rembrandt condition (*p* < 0.001 and *p* = 0.001, respectively) (see Figure 4).

### Trustworthiness

**Figure 5:**
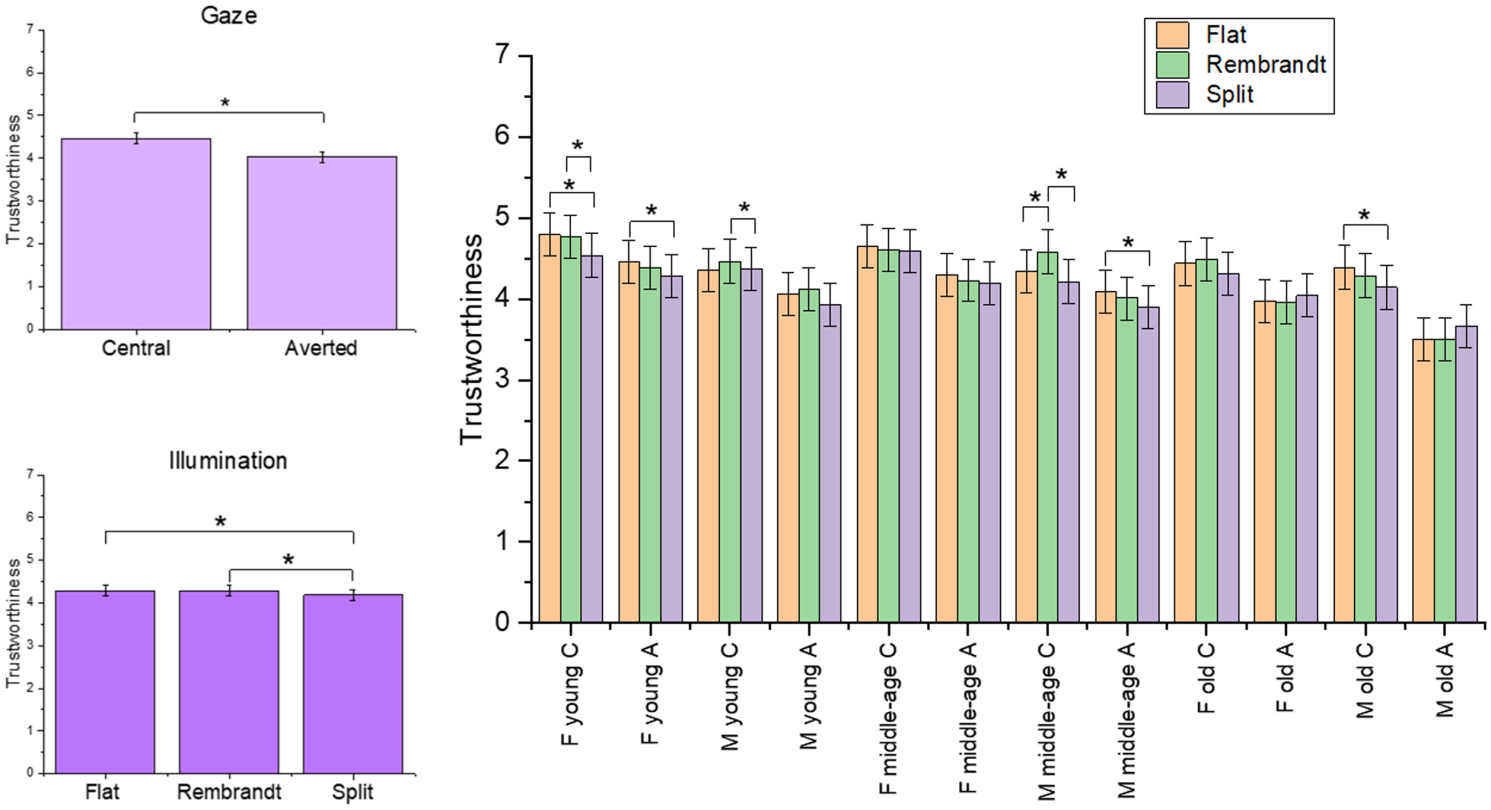
Plot of the estimated mean scores from the trustworthiness rating, depicting significant main effects and higher order interaction. The asterisks highlight the significant main effect and significant results form *post hoc* analyses. C = central gaze, A = averted gaze, F = female models, M = male models. Error bars represent mean standard errors.

The final model on the trustworthiness rating included the four-way interaction [*χ*^2^(4) = 13.24, *p* = 0.01]. The main effects of illumination [*χ*^2^(2) = 31.42, *p* < 0.001] and gaze [*χ*^2^(1) = 829.03, *p* < 0.001] were significant (see Supplementary Table S6 for the model selection). Faces with central gaze were rated more trustworthy than faces with averted gaze, and scores were lower for split compared to flat and Rembrandt illumination (*ps* < 0.001). Contrasts between illumination conditions for the four-way interaction showed that in the case of female models, illumination affected trustworthiness score only in the younger age class, in which scores were lower for split compared to flat (*p* = 0.02) and Rembrandt condition (*p* = 0.04) when faces had central gaze, whereas scores were lower for split compared only to flat (*p* = 0.01) condition when faces had averted gaze. Different modulations were present in male models: faces with split illumination were rated less trustworthy compared to flat illumination in older adults with central gaze (*p* = 0.02) and in middle-age adults with averted gaze (*p* = 0.005); significant lower scores for split compared to Rembrandt illumination resulted in middle-age males with central gaze (*p* < 0.001) and in young males with averted gaze (*p* = 0.007), moreover Rembrandt illumination received higher scores than flat illumination in middle-age males with central gaze (*p* = 0.03) (see Figure 5).

## Discussion

This study presents the Bi-AGI Database, a new set of 30 individual faces with male and female models of different age across lifespan portrayed in three different lighting conditions with central and averted eye gaze. The stimuli were validated with rating questionnaires assessing how illumination conditions and gaze direction, across different ages and gender, affect face evaluation in terms of attractiveness, femininity-masculinity, dominance and trustworthiness. A control experiment was also performed to verify that faces were perceived with neutral expression as expected.

Data from the control experiment confirmed a predominantly neutral perception of faces, although the percentage of happy responses showed that distribution to non-neutral responses was not totally random across the other emotions. This appeared in line with studies adopting a multidimensional perspective, which have shown that the identification of emotional expressions would take place within a dimensional space, rather than within discrete categories, and that neutral expression is located in a space closer to the expression of happiness (Shah and Lewis, 2003).

In line with previous literature, we found that gender, eye gaze, and age influenced face judgments. In particular, as expected, when considering gender and gaze variables, we found that females were rated as more attractive, feminine, trustworthy and less dominant than males (Perrett et al., 2002; Sutherland et al., 2013; Batres et al., 2015), whereas models with central eye gaze were generally judged more attractive, trustworthy and dominant than models with averted gaze (Main et al., 2009; Ewing et al., 2010; Kaisler and Leder, 2016; Mattavelli et al., 2021). Moreover, younger models scored higher on attractiveness (Thornhill and Gangestad, 1999), whereas judgments along the femininity-masculinity dimension increased with increasing age (Batres et al., 2015).

In addition, we investigated whether face judgments were affected by illumination condition, which was varied using three types of photography lighting. Flat, Rembrandt and split lighting conditions were obtained by moving the flash at three different angles from the camera (0°, 75° and 90°, respectively). We considered these settings as they are commonly used in portrait photography to emphasize different facial characteristics or effects conveyed by the pictures (Arena, 2012). Notably, previous studies explored the evaluation of Rembrandt’s paints (from which the lighting features of Rembrandt type portrait were derived) in original or mirror-reversed position, highlighting significant impact of such particular position on cognitive and emotional processing of the stimuli (Schirillo, 2000, 2014; Powell and Schirillo, 2011). However, to the best of our knowledge, there are no previous studies investigating the impact of Rembrandt and split lighting, both consisting in lateralized illumination, on judgment of photographic portrait. Our results showed that these conditions affected evaluations on attractiveness, femininity-masculinity, dominance and trustworthiness of the stimuli, although with different impact in relation to age, gender and gaze of the faces. Split lighting has the ability to create a sharp effect on faces (Grey, 2004), indeed, it overall increased ratings of dominance, while decreased ratings of attractiveness and trustworthiness; furthermore, it increased masculinity in young female with averted gaze.

On the other hand, the Rembrandt illumination is often used in professional photography to give an intense and warm effect to faces (Grey, 2004). The present results support the modulatory role of this type of face illumination using systematic ratings in experimental setting. In particular, compared to split lighting, Rembrandt lighting increased the attractiveness, and trustworthiness rating in young and middle-aged models. Furthermore, this illumination appeared to have consistent effects for dominance and femininity-masculinity judgments related to gender of the stimuli: especially in middle-aged models it reduced the gender characterization of faces increasing dominance and masculinity of female faces and decreasing dominance and masculinity of male faces. This appeared in line with previous evidence showing that lateralized portrait of faces differently affects judgments of male and female models, which was exploited by painters (e.g., Rembrandt) to emphasize gender-related features (Schirillo, 2000). Indeed, males were more often portraited in a right-cheek position to increase social appealing, while females were mainly portraited in left-cheek position to give a demure appearance (Schirillo, 2000). Our models were all photographed with the right-side of the face illuminated and data add further evidence on the interaction between gender and lateralized face presentation. However, further investigation directly comparing right- and left-side illumination are required to support our findings.

These effects of illumination condition support previous evidence concerning within-person variability in face processing and social attribution (Jenkins et al., 2011; Todorov and Porter, 2014) and confirm that light is a critical feature affecting evaluation of ambient pictures in forming first impression (Hill and Bruce, 1996; Burton, 2013). Interestingly, in the analyses of the different ratings we found significant interactions among the factors of interest, suggesting that multiple facial features are considered together to create and evaluate face representations (Thornhill and Gangestad, 1999; Sutherland et al., 2013).

In particular, illumination differently affected attractiveness rating across gender and age: flat was rated more attractive than split condition with young and middle-age females, whereas middle-age males were more attractive with both flat and Rembrandt illumination compared to split; all faces were rated more attractive when posed with a central gaze, but with gender differences associated to age. It is also worth noting that, apart from the effect of split condition on trustworthiness of older males, *post hoc* tests revealed that illumination did not significantly affect rating scores in older adults, suggesting that evaluation of this age class was less influenced by contextual features.

In summary, the present study supports the evidence that face evaluation depends on multiple variables related to individual characteristics, changing aspects of faces and context, which interact to modulate social judgments (Burton, 2013). Our findings add novel data on the role of specific photographic portrait styles, and stimuli from the database could be used in further research exploring the impact of the different types of lateralized illumination on face evaluation. Indeed, the high-quality stimuli will be available for studies of face processing in a wide range of research fields from experimental psychology, perception, or face morphing to computational modeling studies for training face recognition algorithms (Adini et al., 1997; Georghiades et al., 2001). Moreover, the natural manipulation of gaze direction provides new stimuli ready to be used in joint attention paradigms, thus sparing the time-consuming editing procedure, and ensuring the ecological validity of stimuli depicting faces of different ages.

In conclusion, the Bi-AGI database offers the advantages of freely available face stimuli with manipulation of illumination in photographic settings and natural gaze directions, covering a wide age range across lifespan. The average ratings for each individual model are provided in the Appendix A, facilitating the selection of faces with particular characteristics and making Bi-AGI a feasible new tool for the scientific community.

## Data availability statement

The original contributions presented in the study are included in the article/Supplementary material, further inquiries can be directed to the corresponding author. Stimuli of the Bi-AGI database are available upon request at: https://board.unimib.it/datasets/rx6kpwmvtf/1.

## Ethics statement

The studies involving human participants were reviewed and approved by Ethic Committee of the University Milano-Bicocca. The patients/participants provided their written informed consent to participate in this study. Written informed consent was obtained from the individual(s) for the publication of any identifiable images or data included in this article.

## Author contributions

GM and PR contributed to conception and design of the study. EC organized the database. IG and JD performed the statistical analysis. GM wrote the first draft of the manuscript. IG and JD wrote sections of the manuscript. All authors contributed to the article and approved the submitted version.

## Funding

The study was funded by a grant from University of Milano-Bicocca to PR. IG was partially supported by grants from Italian Ministry of Health to GM (GR-2016-02361283).

## Acknowledgments

The authors thank Radice, Stifanelli, and Scordamaglia for their help in data collection.

## Conflict of interest

The authors declare that the research was conducted in the absence of any commercial or financial relationships that could be construed as a potential conflict of interest.

## Publisher’s note

All claims expressed in this article are solely those of the authors and do not necessarily represent those of their affiliated organizations, or those of the publisher, the editors and the reviewers. Any product that may be evaluated in this article, or claim that may be made by its manufacturer, is not guaranteed or endorsed by the publisher.
