# Supplementary material for: Illumination and gaze effects on face evaluation: The Bi-AGI database
Source: Front Psychol. 2022 Oct 13;13:948142. doi: 10.3389/fpsyg.2022.948142 (PMC9608625; doi:10.3389/fpsyg.2022.948142)
Supplement: Supplementary file 1 [file Table_1.DOCX]

**Supplementary material for:**

**Illumination and gaze effects on face evaluation: the Bi-AGI Database**

G. Mattavelli, I. Gorrino, E. Cesana, J. De Angelis, P. Ricciardelli

**Supplementary Tables on control experiment**

Table S1. RESPs real and expected distribution – Chi Square test.

| Emotion (RESPs) | Real Distribution | Expected distribution (equidistribution) |
| --- | --- | --- |
| 1. Neutral | 30.40 % | 14.28 % |
| 1. Surprise | 10,01 % | 14.28 % |
| 1. Happiness | 26,66 % | 14.28 % |
| 1. Fear | 8,30 % | 14.28 % |
| 1. Disgust | 6,49 % | 14.28 % |
| 1. Anger | 6,86 % | 14.28 % |
| 1. Sadness | 11,20 % | 14.28 % |

Table S2. RESPs real and expected RESPs pairs % - binomial test.

| RESPs pair | Real % of neutral responses | Expected % of neutral responses | Binomial test significance value |
| --- | --- | --- | --- |
| Neutral-Surprise | 81,66 % | 50 % | < .0001 |
| Neutral-Happiness | 53,31 % | 50 % | 0.21 |
| Neutral-Fear | 78,56 % | 50 % | < .0001 |
| Neutral-Disgust | 82,43 % | 50 % | < .0001 |
| Neutral-Anger | 81,61 % | 50 % | < .0001 |
| Neutral-Sadness | 73,10 % | 50 % | < .0001 |

**Validation study models selection**

Table S3: Likelihood ratio tests procedure for model selection on attractiveness rating.

| *Fixed factors* | df | χ^2^ | p |
| --- | --- | --- | --- |
| Illumination | 2 | 23.33 | <.001 |
| Gender | 1 | 11 | <.001 |
| Age | 2 | 39.7 | <.001 |
| Gaze | 1 | 169.42 | <.001 |
| Illumination * Gender | 2 | 4.32 | .11 |
| Illumination * Age | 4 | 16.59 | .002 |
| Gender * Age | 2 | 6.44 | .039 |
| Gender * Gaze | 1 | 2.42 | .0119 |
| Age * Gaze | 2 | 7.78 | .020 |
| Illumination * Gender * Age | 4 | 12.7 | .012 |
| Gender *Age * Gaze | 2 | 6.19 | .045 |
| *Random effects* |  |  |  |
| 1\|Subect | 1 | 15134 | <.001 |
| 1\|Face model | 1 | 4029.8 | <.001 |

Table S3 summarizes the model-simplification procedure, including degrees of freedom, chi square and significance. Fixed factors were not removed when they were part of higher order interactions.

Table S4: Likelihood ratio tests procedure for model selection on femininity-masculinity rating.

| *Fixed factors* | df | χ^2^ | p |
| --- | --- | --- | --- |
| Illumination | 2 | 6.03 | .049 |
| Gaze | 1 | 0.001 | .98 |
| Gender | 1 | 726.17 | <.001 |
| Age | 2 | 21.87 | <.001 |
| Illumination * Gaze | 2 | 0.47 | .79 |
| Illumination * Gender | 2 | 62.82 | <.001 |
| Gaze * Gender | 1 | 21.35 | <.001 |
| Illumination * Age | 4 | 3.78 | .44 |
| Gaze * Age | 2 | 0.82 | .66 |
| Gender * Gaze | 2 | 0.04 | .98 |
| Illumination * Gaze * Gender | 2 | 66.09 | <.001 |
| Illumination * Gaze * Age | 4 | 5.39 | .25 |
| Illumination * Gender * Age | 4 | 103.87 | <.001 |
| Gaze * Gender * Age | 2 | 63.33 | <.001 |
| Illumination * Gaze * Gender * Age | 4 | 154.14 | <.001 |
| *Random effects* |  |  |  |
| 1\|Subect | 1 | 4127 | <.001 |
| 1\|Face model | 1 | 1739.4 | <.001 |

Table S4 summarizes the model-simplification procedure, including degrees of freedom, chi square and significance. Fixed factors were not removed when they were part of higher order interactions.

Table S5: Likelihood ratio tests procedure for model selection on dominance rating.

| *Fixed factors* | df | χ^2^ | p |
| --- | --- | --- | --- |
| Illumination | 2 | 11.58 | .003 |
| Gaze | 1 | 31 | <.001 |
| Gender | 1 | 1.43 | .23 |
| Age | 2 | 4.6 | .10 |
| Illumination * Gaze | 2 | 0.36 | .83 |
| Illumination * Gender | 2 | 8.08 | .017 |
| Gaze * Gender | 1 | 5.04 | .024 |
| Illumination * Age | 4 | 10.17 | .037 |
| Gaze * Age | 2 | 20.4 | <.001 |
| Gender * Gaze | 2 | 0.73 | .69 |
| Illumination * Gaze * Gender | 2 | 2.58 | .27 |
| Illumination * Gaze * Age | 4 | 3.40 | .49 |
| Illumination * Gender * Age | 4 | 17.38 | <.001 |
| Gaze * Gender * Age | 2 | 1.07 | .586 |
| Illumination * Gaze * Gender * Age | 4 | 40.63 | <.001 |
| *Random effects* |  |  |  |
| 1\|Subect | 1 | 6694.8 | <.001 |
| 1\|Face model | 1 | 6526.2 | <.001 |

Table S5 summarizes the model-simplification procedure, including degrees of freedom, chi square and significance. Fixed factors were not removed when they were part of higher order interactions.

Table S6: Likelihood ratio tests procedure for model selection on trustworthiness rating.

| *Fixed factors* | df | χ^2^ | p |
| --- | --- | --- | --- |
| Illumination | 2 | 31.42 | <.001 |
| Gaze | 1 | 829.03 | <.001 |
| Gender | 1 | 2.1 | .14 |
| Age | 2 | 2.21 | .33 |
| Illumination * Gaze | 2 | 14 | <.001 |
| Illumination * Gender | 2 | 25 | .25 |
| Gaze * Gender | 1 | 44.26 | <.001 |
| Illumination * Age | 4 | 0.04 | <.001 |
| Gaze * Age | 2 | 44.26 | <.001 |
| Gender * Gaze | 2 | 0.04 | .98 |
| Illumination * Gaze * Gender | 2 | 0.3 | .86 |
| Illumination * Gaze * Age | 4 | 23.32 | <.001 |
| Illumination * Gender * Age | 4 | 12.77 | .012 |
| Gaze * Gender * Age | 2 | 19.07 | <.001 |
| Illumination * Gaze * Gender * Age | 4 | 13.24 | .010 |
| *Random effects* |  |  |  |
| 1\|Subect | 1 | 10013 | <.001 |
| 1\|Face model | 1 | 4676.3 | <.001 |

Table S6 summarizes the model-simplification procedure, including degrees of freedom, chi square and significance. Fixed factors were not removed when they were part of higher order interactions.
