# Supplementary material for: Illumination and gaze effects on face evaluation: The Bi-AGI database
Source: Front Psychol. 2022 Oct 13;13:948142. doi: 10.3389/fpsyg.2022.948142 (PMC9608625; doi:10.3389/fpsyg.2022.948142)
Supplement: Supplementary file 2 [file Table_2.docx]

**Appendix A**

Mean rating values for each stimulus separately in the different illumination and gaze conditions (C=central, A=averted). Models’ code corresponds with file name of images within the database, age is reported in years with the corresponding age cohort within brackets (Y=young, M=middle-age, O=older adults).

|  | | Attractiveness | | | | | | Gender | | | | | | Dominance | | | | | | Trustworthiness | | | | | |
| --- | --- | --- | --- | --- | --- | --- | --- | --- | --- | --- | --- | --- | --- | --- | --- | --- | --- | --- | --- | --- | --- | --- | --- | --- | --- |
|  | | Flat | | Rembrandt | | Split | | Flat | | Rembrandt | | Split | | Flat | | Rembrandt | | Split | | Flat | | Rembrandt | | Split | |
| Model | Age | C | A | C | A | C | A | C | A | C | A | C | A | C | A | C | A | C | A | C | A | C | A | C | A |
| F1 | 25 (Y) | 5.10 | 5.09 | 5.14 | 4.98 | 4.98 | 5.00 | 3.03 | 2.88 | 3.07 | 2.85 | 3.17 | 3.05 | 4.64 | 4.17 | 4.46 | 4.29 | 4.52 | 4.34 | 4.79 | 4.81 | 5.04 | 4.70 | 4.79 | 4.50 |
| F2 | 18 (Y) | 3.54 | 3.36 | 3.47 | 3.43 | 3.39 | 3.22 | 5.33 | 5.39 | 5.34 | 5.37 | 5.35 | 5.39 | 3.39 | 3.18 | 3.34 | 3.18 | 3.60 | 3.55 | 4.70 | 4.20 | 4.81 | 4.23 | 4.33 | 3.76 |
| F3 | 19 (Y) | 5.35 | 5.03 | 5.10 | 4.74 | 4.91 | 4.74 | 1.82 | 1.95 | 2.19 | 2.15 | 2.11 | 2.19 | 2.83 | 3.08 | 3.18 | 3.06 | 3.13 | 2.84 | 5.67 | 5.01 | 5.17 | 4.96 | 5.26 | 4.90 |
| F4 | 23 (Y) | 3.07 | 2.81 | 3.19 | 3.10 | 3.10 | 2.91 | 5.79 | 5.79 | 5.70 | 5.80 | 5.90 | 5.92 | 4.37 | 4.23 | 4.32 | 4.27 | 4.45 | 4.39 | 3.89 | 3.64 | 4.16 | 3.77 | 3.96 | 3.43 |
| F5 | 19 (Y) | 5.65 | 5.51 | 5.70 | 5.47 | 5.59 | 5.35 | 1.76 | 1.72 | 1.80 | 1.76 | 1.81 | 1.87 | 3.21 | 3.16 | 3.30 | 3.13 | 3.42 | 3.13 | 5.53 | 5.26 | 5.69 | 5.15 | 5.33 | 5.04 |
| F6 | 39 (M) | 2.47 | 2.43 | 2.73 | 2.71 | 2.79 | 2.66 | 4.79 | 4.85 | 4.93 | 4.80 | 4.72 | 4.76 | 2.90 | 2.73 | 2.92 | 2.84 | 2.71 | 2.79 | 3.76 | 3.55 | 3.98 | 3.76 | 3.75 | 3.61 |
| F7 | 35(M) | 4.24 | 4.10 | 4.36 | 4.21 | 3.98 | 3.84 | 2.72 | 2.79 | 2.71 | 2.74 | 2.90 | 2.89 | 4.77 | 4.56 | 4.46 | 4.41 | 4.77 | 4.71 | 4.00 | 3.63 | 4.17 | 3.66 | 3.67 | 3.40 |
| F8 | 35 (M) | 3.92 | 3.84 | 3.61 | 3.51 | 3.82 | 3.66 | 5.74 | 5.82 | 5.82 | 5.74 | 5.64 | 5.57 | 3.62 | 3.53 | 3.76 | 3.53 | 3.58 | 3.51 | 4.90 | 4.63 | 4.61 | 4.32 | 4.87 | 4.48 |
| F9 | 35 (M) | 3.58 | 3.28 | 3.27 | 3.25 | 3.39 | 3.34 | 2.46 | 2.51 | 2.53 | 2.63 | 2.57 | 2.57 | 2.59 | 2.65 | 2.68 | 2.64 | 2.79 | 2.70 | 3.99 | 3.58 | 3.79 | 3.45 | 3.64 | 3.53 |
| F10 | 43 (M) | 3.87 | 3.82 | 4.10 | 4.07 | 4.16 | 3.96 | 5.79 | 5.64 | 5.77 | 5.61 | 5.70 | 5.66 | 4.03 | 4.06 | 4.16 | 3.80 | 4.09 | 4.17 | 4.56 | 4.33 | 4.76 | 4.56 | 4.97 | 4.38 |
| F11 | 67 (O) | 4.56 | 4.32 | 4.08 | 4.00 | 3.88 | 3.76 | 2.73 | 2.83 | 2.69 | 2.89 | 3.20 | 3.01 | 4.59 | 4.64 | 4.64 | 4.58 | 4.78 | 4.78 | 4.28 | 3.73 | 4.10 | 3.69 | 3.76 | 3.42 |
| F12 | 63 (O) | 3.54 | 3.29 | 3.26 | 3.11 | 3.39 | 3.18 | 5.92 | 5.80 | 5.72 | 5.79 | 5.78 | 5.74 | 3.30 | 3.26 | 3.43 | 3.30 | 3.22 | 3.16 | 5.24 | 5.01 | 5.06 | 4.82 | 4.84 | 4.71 |
| F13 | 86 (O) | 3.56 | 3.32 | 3.73 | 3.45 | 3.46 | 3.41 | 2.69 | 2.67 | 2.61 | 2.69 | 2.57 | 2.67 | 3.02 | 2.81 | 2.98 | 2.81 | 2.98 | 2.81 | 5.34 | 4.83 | 5.16 | 4.87 | 5.11 | 4.86 |
| F14 | 84 (O) | 3.95 | 3.77 | 3.91 | 3.87 | 3.73 | 3.51 | 6.12 | 5.97 | 5.98 | 5.95 | 6.06 | 6.00 | 4.47 | 4.24 | 4.56 | 4.38 | 4.74 | 4.43 | 4.74 | 4.53 | 4.53 | 4.50 | 4.24 | 4.07 |
| F15 | 72 (O) | 3.91 | 3.70 | 3.90 | 3.70 | 3.72 | 3.61 | 2.65 | 2.56 | 2.65 | 2.63 | 2.50 | 2.66 | 4.13 | 3.99 | 3.97 | 3.78 | 4.18 | 4.16 | 4.30 | 4.13 | 4.37 | 4.04 | 4.53 | 4.03 |
| M1 | 25 (Y) | 2.56 | 2.28 | 2.63 | 2.21 | 2.59 | 2.13 | 5.61 | 5.55 | 5.53 | 5.53 | 5.53 | 5.53 | 3.24 | 3.14 | 3.44 | 3.14 | 3.52 | 3.36 | 3.33 | 2.89 | 3.72 | 2.83 | 3.45 | 2.81 |
| M2 | 25 (Y) | 3.77 | 3.81 | 3.98 | 3.59 | 4.14 | 3.90 | 2.55 | 2.54 | 6.01 | 2.61 | 2.50 | 2.53 | 3.42 | 3.26 | 4.39 | 3.21 | 3.44 | 3.39 | 4.30 | 4.10 | 4.76 | 4.03 | 4.90 | 4.31 |
| M3 | 25 (Y) | 3.83 | 3.57 | 4.15 | 3.63 | 3.59 | 3.53 | 6.30 | 6.32 | 2.29 | 6.14 | 6.29 | 6.18 | 5.24 | 4.99 | 3.01 | 4.64 | 4.92 | 4.64 | 4.29 | 3.74 | 5.21 | 3.93 | 4.33 | 4.03 |
| M4 | 21 (Y) | 3.64 | 3.37 | 3.41 | 3.27 | 3.14 | 3.23 | 2.76 | 2.70 | 2.89 | 2.90 | 3.04 | 2.91 | 3.50 | 3.27 | 3.96 | 3.43 | 3.78 | 3.64 | 5.01 | 4.73 | 4.67 | 4.52 | 4.69 | 4.37 |
| M5 | 19 (Y) | 3.16 | 3.29 | 3.16 | 3.10 | 3.15 | 2.96 | 6.12 | 5.96 | 6.07 | 5.93 | 6.11 | 6.05 | 4.73 | 3.92 | 4.83 | 4.42 | 4.43 | 4.44 | 4.13 | 4.30 | 4.41 | 3.97 | 4.21 | 3.86 |
| M6 | 38 (M) | 2.80 | 2.48 | 2.64 | 2.34 | 2.68 | 2.40 | 6.05 | 6.02 | 6.14 | 6.07 | 6.11 | 6.06 | 4.07 | 4.50 | 4.59 | 4.61 | 4.54 | 4.59 | 4.63 | 3.84 | 4.35 | 3.77 | 4.39 | 3.96 |
| M7 | 35 (M) | 2.50 | 2.42 | 2.72 | 2.51 | 2.55 | 2.38 | 3.37 | 3.35 | 3.27 | 3.39 | 3.37 | 3.43 | 4.26 | 4.30 | 4.10 | 4.31 | 4.34 | 4.19 | 4.13 | 3.66 | 4.27 | 3.74 | 3.89 | 3.69 |
| M8 | 39 (M) | 3.04 | 2.60 | 3.09 | 2.68 | 2.88 | 2.90 | 6.11 | 6.18 | 6.16 | 6.10 | 6.11 | 6.12 | 4.83 | 4.61 | 4.75 | 4.33 | 4.76 | 4.66 | 4.90 | 4.17 | 4.96 | 4.16 | 4.76 | 4.69 |
| M9 | 42 (M) | 3.13 | 2.74 | 3.15 | 2.82 | 3.11 | 2.76 | 2.87 | 3.16 | 3.06 | 3.17 | 3.10 | 3.13 | 4.96 | 4.93 | 4.88 | 4.96 | 4.59 | 4.71 | 4.24 | 3.43 | 4.19 | 3.44 | 4.17 | 3.66 |
| M10 | 44 (M) | 2.56 | 2.42 | 2.76 | 2.47 | 2.45 | 2.21 | 6.22 | 6.33 | 6.20 | 6.26 | 6.26 | 6.33 | 4.88 | 5.29 | 5.07 | 5.16 | 5.26 | 5.49 | 4.19 | 3.30 | 4.19 | 3.31 | 3.69 | 3.16 |
| M11 | 87 (O) | 2.61 | 2.55 | 2.93 | 2.47 | 2.67 | 2.75 | 3.00 | 2.93 | 2.88 | 2.97 | 2.96 | 2.96 | 3.39 | 3.07 | 3.06 | 3.24 | 3.24 | 3.00 | 4.86 | 4.60 | 5.02 | 4.36 | 4.68 | 4.61 |
| M12 | 79 (M) | 2.87 | 2.14 | 2.94 | 2.27 | 2.71 | 2.31 | 5.73 | 5.66 | 5.76 | 5.70 | 5.73 | 5.63 | 3.19 | 3.44 | 3.20 | 3.27 | 3.45 | 3.16 | 4.98 | 3.28 | 4.82 | 3.53 | 4.64 | 3.66 |
| M13 | 55 (M) | 2.52 | 2.38 | 2.71 | 2.47 | 2.71 | 2.48 | 3.12 | 3.10 | 3.06 | 3.17 | 2.97 | 3.13 | 4.39 | 4.37 | 4.09 | 4.14 | 3.64 | 3.94 | 4.43 | 3.94 | 4.55 | 3.94 | 4.59 | 3.97 |
| M14 | 64 (O) | 2.41 | 2.17 | 2.24 | 2.25 | 2.30 | 2.23 | 6.24 | 6.27 | 6.36 | 6.34 | 6.30 | 6.31 | 5.10 | 5.00 | 5.30 | 5.24 | 4.98 | 5.36 | 3.27 | 2.94 | 3.12 | 2.77 | 3.24 | 2.86 |
| M15 | 60 (O) | 2.67 | 2.67 | 2.57 | 2.65 | 2.55 | 2.48 | 2.93 | 2.91 | 3.12 | 2.97 | 3.09 | 2.99 | 3.36 | 3.43 | 3.70 | 3.41 | 3.90 | 3.40 | 4.54 | 4.23 | 4.44 | 4.35 | 4.24 | 4.29 |

**Appendix B**

List of pictures in the database for each model.

Database link: https://board.unimib.it/datasets/rx6kpwmvtf/1

| Model | Flat | | | Rembrandt | | | Split | | |
| --- | --- | --- | --- | --- | --- | --- | --- | --- | --- |
|  | C | A | | C | A | | C | A | |
|  |  | L | R |  | L | R |  | L | R |
| F1 | file1.jpg | file3.jpg | file5.jpg | file7.jpg | file9.jpg | file11.jpg | file13.jpg | file15.jpg | file17.jpg |
| F2 | file19.jpg | file21.jpg | file23.jpg | file25.jpg | file27.jpg | file29.jpg | file31.jpg | file33.jpg | file35.jpg |
| F3 | file37.jpg | file39.jpg | file41.jpg | file43.jpg | file45.jpg | file47.jpg | file49.jpg | file51.jpg | file53.jpg |
| F4 | file55.jpg | file57.jpg | file59.jpg | file61.jpg | file63.jpg | file65.jpg | file67.jpg | file69.jpg | file71.jpg |
| F5 | file73.jpg | file75.jpg | file77.jpg | file79.jpg | file81.jpg | file83.jpg | file85.jpg | file87.jpg | file89.jpg |
| F6 | file91.jpg | file93.jpg | file95.jpg | file97.jpg | file99.jpg | file101.jpg | file103.jpg | file105.jpg | file107.jpg |
| F7 | file109.jpg | file111.jpg | file113.jpg | file115.jpg | file117.jpg | file119.jpg | file121.jpg | file123.jpg | file125.jpg |
| F8 | file127.jpg | file129.jpg | file131.jpg | file133.jpg | file135.jpg | file137.jpg | file139.jpg | file141.jpg | file143.jpg |
| F9 | file145.jpg | file147.jpg | file149.jpg | file151.jpg | file153.jpg | file155.jpg | file157.jpg | file159.jpg | file161.jpg |
| F10 | file163.jpg | file165.jpg | file167.jpg | file169.jpg | file171.jpg | file173.jpg | file175.jpg | file177.jpg | file179.jpg |
| F11 | file182.jpg | file184.jpg | file186.jpg | file188.jpg | file190.jpg | file192.jpg | file194.jpg | file196.jpg | file198.jpg |
| F12 | file200.jpg | file202.jpg | file204.jpg | file206.jpg | file208.jpg | file210.jpg | file212.jpg | file214.jpg | file216.jpg |
| F13 | file218.jpg | file220.jpg | file222.jpg | file224.jpg | file226.jpg | file228.jpg | file230.jpg | file232.jpg | file234.jpg |
| F14 | file236.jpg | file238.jpg | file240.jpg | file242.jpg | file244.jpg | file246.jpg | file248.jpg | file250.jpg | file252.jpg |
| F15 | file254.jpg | file256.jpg | file258.jpg | file260.jpg | file262.jpg | file264.jpg | file266.jpg | file268.jpg | file270.jpg |
| M1 | file2.jpg | file4.jpg | file6.jpg | file8.jpg | file10.jpg | file12.jpg | file14.jpg | file16.jpg | file18.jpg |
| M2 | file20.jpg | file22.jpg | file24.jpg | file26.jpg | file28.jpg | file30.jpg | file32.jpg | file34.jpg | file36.jpg |
| M3 | file38.jpg | file40.jpg | file42.jpg | file44.jpg | file46.jpg | file48.jpg | file50.jpg | file52.jpg | file54.jpg |
| M4 | file56.jpg | file58.jpg | file60.jpg | file62.jpg | file64.jpg | file66.jpg | file68.jpg | file70.jpg | file72.jpg |
| M5 | file74.jpg | file76.jpg | file78.jpg | file80.jpg | file82.jpg | file84.jpg | file86.jpg | file88.jpg | file90.jpg |
| M6 | file92.jpg | file94.jpg | file96.jpg | file98.jpg | file100.jpg | file102.jpg | file104.jpg | file106.jpg | file108.jpg |
| M7 | file110.jpg | file112.jpg | file114.jpg | file116.jpg | file118.jpg | file120.jpg | file122.jpg | file124.jpg | file126.jpg |
| M8 | file128.jpg | file130.jpg | file132.jpg | file134.jpg | file136.jpg | file138.jpg | file140.jpg | file142.jpg | file144.jpg |
| M9 | file146.jpg | file148.jpg | file150.jpg | file152.jpg | file154.jpg | file156.jpg | file158.jpg | file160.jpg | file162.jpg |
| M10 | file164.jpg | file166.jpg | file168.jpg | file170.jpg | file172.jpg | file174.jpg | file176.jpg | file178.jpg | file180.jpg |
| M11 | file181.jpg | file183.jpg | file185.jpg | file187.jpg | file189.jpg | file191.jpg | file193.jpg | file195.jpg | file197.jpg |
| M12 | file199.jpg | file201.jpg | file203.jpg | file205.jpg | file207.jpg | file209.jpg | file211.jpg | file213.jpg | file215.jpg |
| M13 | file217.jpg | file219.jpg | file221.jpg | file223.jpg | file225.jpg | file227.jpg | file229.jpg | file231.jpg | file233.jgp |
| M14 | file235.jpg | file237.jpg | file239.jpg | file241.jpg | file243.jpg | file245.jpg | file247.jpg | file249.jpg | file251.jpg |
| M15 | file253.jpg | file255.jpg | file257.jpg | file259.jpg | file261.jpg | file263.jpg | file265.jpg | file267.jpg | file269.jpg |
